# Calcium-Regulated Phosphorylation Systems Controlling Uptake and Balance of Plant Nutrients

**DOI:** 10.3389/fpls.2020.00044

**Published:** 2020-02-11

**Authors:** Shunya Saito, Nobuyuki Uozumi

**Affiliations:** Department of Biomolecular Engineering, Graduate School of Engineering, Tohoku University, Sendai, Japan

**Keywords:** nutrition, calcium, membrane transport, *Arabidopsis thaliana*, ion homeostasis

## Abstract

Essential elements taken up from the soil and distributed throughout the whole plant play diverse roles in different tissues. Cations and anions contribute to maintenance of intracellular osmolarity and the formation of membrane potential, while nitrate, ammonium, and sulfate are incorporated into amino acids and other organic compounds. In contrast to these ion species, calcium concentrations are usually kept low in the cytosol and calcium displays unique behavior as a cytosolic signaling molecule. Various environmental stresses stimulate increases in the cytosolic calcium concentration, leading to activation of calcium-regulated protein kinases and downstream signaling pathways. In this review, we summarize the stress responsive regulation of nutrient uptake and balancing by two types of calcium-regulated phosphorylation systems: CPK and CBL-CIPK. CPK is a family of protein kinases activated by calcium. CBL is a group of calcium sensor proteins that interact with CIPK kinases, which phosphorylate their downstream targets. In *Arabidopsis*, quite a few ion transport systems are regulated by CPKs or CBL-CIPK complexes, including channels/transporters that mediate transport of potassium (KAT1, KAT2, GORK, AKT1, AKT2, HAK5, SPIK), sodium (SOS1), ammonium (AMT1;1, AMT1;2), nitrate and chloride (SLAC1, SLAH2, SLAH3, NRT1.1, NRT2.4, NRT2.5), and proton (AHA2, V-ATPase). CPKs and CBL-CIPKs also play a role in C/N nutrient response and in acquisition of magnesium and iron. This functional regulation by calcium-dependent phosphorylation systems ensures the growth of plants and enables them to acquire tolerance against various environmental stresses. Calcium serves as the key factor for the regulation of membrane transport systems.

## Introduction

Plants require various ions as essential nutrients, which are taken up from the soil and distributed throughout the whole plant (Welch, 1995; Merchant, 2010; Grusak et al., 2016). Each of these nutrients, once they are transferred to their destination within plant tissues *via* corresponding transporters/ion channels, plays diverse and critical roles in maintaining plant growth. Potassium, nitrate, and chloride contribute to maintenance of intracellular osmolarity, enabling control of cell turgor pressure which is crucial for cell expansion, stomatal movement, and pollen tube growth (Kroeger et al., 2011; Saito and Uozumi, 2019). Nitrate, ammonium, sulfate, and phosphorus are metabolized to produce various proteins and organic compounds (Leustek and Saito, 1999; López-Arredondo et al., 2013; López-Arredondo et al., 2014). Metal ions such as iron (Balk and Schaedler, 2014), manganese (Schmidt et al., 2016; Schmidt and Husted, 2019), magnesium (Gerendás and Führs, 2013), zinc (Broadley et al., 2007) and molybdenum (Mendel, 2013) work as essential cofactors for enzyme activity.

Among these essential nutrient ions, calcium exhibits some unique behaviors. In contrast to other macronutrient ions, such as potassium, for which the cellular concentration is normally in the range of 80 to 100 mM, calcium concentrations are usually relatively low and kept around 0.1 μM in the cytosol (Bush, 1995; Walker et al., 1996; Sanders et al., 1999; Hepler, 2005). However, when plants are exposed to environmental stresses such as drought, saline soil, pathogens, wounding, or nutrient deficiency, a rapid increase of the cytosolic calcium concentration occurs, either as a result of Ca^2+^ import *via* plasma membrane ion channels or Ca^2+^ release from intracellular calcium stores (Steinhorst and Kudla, 2013b; Zhu, 2016; Manishankar et al., 2018; Toyota et al., 2018). This leads to activation of calcium-regulated protein kinases, initiation of downstream phosphorylation signaling, and finally, achievement of stress resistance resulting from an activation of stress responsive genes or adjustment of ion channel activity. Ca^2+^-regulated proteins which play a key role in this phosphorylation process can be divided into three major groups: Calcium dependent protein kinases (CPK), CPK-related protein kinases (CRK), and Calcineurin-B like proteins (CBL). CPK is a family of Ser/Thr kinases containing a calcium binding site (EF hand) in their C-terminal region. Binding of Ca^2+^ to the EF hand stimulates a conformational change, thus allowing autophosphorylation of the kinase (Hashimoto and Kudla, 2011; Schulz et al., 2013). There are 34 CPK members in the *Arabidopsis* genome, and over half of these have been functionally characterized (Kudla et al., 2010; Boudsocq and Sheen, 2013; Shi et al., 2018; Saito and Uozumi, 2019). CRKs, on the other hand, were recently shown to be able to phosphorylate Tyr residues (Nemoto et al., 2015). The function of only two of eight CRK members in *Arabidopsis* has been analyzed so far (Rigó et al., 2013; Baba et al., 2018). CBL differs from the other two groups with regard to CBL itself being a Ca^2+^ sensor protein but not a kinase. Ca^2+^-bound and activated CBL interacts with another group of kinases called CBL-interacting protein kinases (CIPK), thereby enhancing CIPK autophosphorylation and recruitment to their target proteins (Batistic et al., 2008; Batistič and Kudla, 2009; Mao et al., 2016). Ten members of CBL and 26 members of CIPK exist in the *Arabidopsis* genome, each has a unique expression and subcellular localization profile. Together they form a specific interaction network, allowing regulation of genes and ion channels in various locations (Mahajan et al., 2006; Steinhorst and Kudla, 2013b; Manik et al., 2015; Manishankar et al., 2018; Saito and Uozumi, 2019). In this review, we focus on the stress responsive regulation of nutrient uptake and balancing by CPK and CBL-CIPK.

### Calcium-Dependent Import of Potassium and Anions—Regulator of Intracellular Osmolarity

Potassium (K^+^) is the most abundant ion in plant cells. As a soluble ion, it plays a critical role in adjusting cellular osmolarity, membrane electric potential, or intracellular pH (Almeida et al., 2017; Ragel et al., 2019). These processes are important for the regulation of cell expansion, which is a prerequisite for plant growth and stomatal movement. Other ion species that contribute to this regulation are nitrate (NO_3_^−^) and chloride (Cl^−^). These anions work synergistically with K^+^ in the regulation of guard cell turgor pressure, and ultimately the control of stomatal aperture. K^+^, NO_3_^−^, and Cl^−^ fluxes across the plasma membrane of pollen tubes are also essential for its growth (Mouline et al., 2002; Wu et al., 2011; Gutermuth et al., 2013; Liu et al., 2016).

Early studies proposed a correlation between cytosolic calcium and the uptake of potassium in a variety of plant species (Hirata and Mitsui, 1965; Johansen et al., 1968; Rains and Floyd, 1970). Indeed, it has been reported that K^+^ deficiency induces rapid Ca^2+^ increase in *Arabidopsis* roots (Behera et al., 2016). In *Arabidopsis* root cells, K^+^ uptake from the soil and export to the xylem are orchestrated by several types of transporters. Main contributors to root K^+^ uptake are the Shaker-type K^+^ channel AKT1 and the KT/KUP/HAK type transporter HAK5 (Pyo et al., 2010; Rubio et al., 2010; Alemán et al., 2011). The activity of these two K^+^ transport systems depends on CBL1 (or CBL9) and CIPK23 (Xu et al., 2006; Lee et al., 2007; Ragel et al., 2015). When cytosolic Ca^2+^ increases, activated CBL1/9 interacts with and recruits CIPK23 to the plasma membrane, enabling it to activate AKT1 and HAK5. Another CBL member, CBL10, is capable of CIPK-independent negative regulation of AKT1 activity, suggesting a role in maintaining balance of K^+^ uptake (Ren et al., 2013). CIPK9, most likely paired with CBL2 or CBL3, also regulates K^+^ homeostasis under low K^+^ conditions *via* phosphorylation of a yet unknown target (Pandey et al., 2007a; Liu et al., 2012b; Singh et al., 2018). In addition, members of the cyclic-nucleotide gated channel family CNGC3, CNGC10, and CNGC13 (Kaplan et al., 2007; Caballero et al., 2012; Ragel et al., 2019), and the cation-proton antiporter CHX13 (Zhao et al., 2008) have also been reported to mediate K^+^ flux into root cell. Activity of these CNGCs might be regulated by Ca^2+^-activated calmodulin binding and resulting blocking of the cyclic-nucleotide binding domain (Kaplan et al., 2007; DeFalco et al., 2016; Pan et al., 2019). K^+^ uptake by AKT1 and HAK5 is also conserved in rice, although systems corresponding to CNGCs and CHX remain to be identified in rice (Ragel et al., 2019). Increase of the K^+^ concentration in root stellar cell enables drive of K^+^ into the xylem mediated by the Shaker-type K^+^ efflux channel SKOR, followed by translocation of K^+^ to the shoot (Liu et al., 2006; Ragel et al., 2019).

In contrast to its role connected to potassium, the role of calcium as a second messenger for the nitrate response was only recently discovered (Riveras et al., 2015). Nitrate uptake and distribution throughout the plant is mainly mediated by members of the nitrate transporter (NRT) or nitrate transporter 1/peptide transporter (NPF) family (Léran et al., 2014). In *Arabidopsis* roots, NRT2.1/2.2/2.4/2.5 and NPF2.3/4.6/6.3 are responsible for NO_3_^−^ uptake and translocation (Taochy et al., 2015; Noguero and Lacombe, 2016; Xuan et al., 2017; Zhao et al., 2018a). Among these transporters, NPF6.3, also known as NRT1.1 or CHL1, is well studied and considered a major contributor to NO_3_^−^ transport (Léran et al., 2013; Leran et al., 2015; Undurraga et al., 2017). NPF6.3 is characterized as a dual affinity bidirectional NO_3_^−^ transporter. This unique transporter switches its affinity from low-affinity to high-affinity mode by dimerization, which is controlled by phosphorylation of Thr101 by CBL1/9-CIPK23 (Liu and Tsay, 2003; Ho et al., 2009; Parker and Newstead, 2014; Sun et al., 2014). Two other members of CBL-CIPK are also involved in regulation of NRT/NPF. CIPK8 plays a role in the nitrate response by influencing the expression level of several nitrate-responsive genes including NPF6.3 and NRT2.1 (Hu et al., 2009). CBL7, on the other hand, was shown to regulate the expression levels of NRT2.4 and NRT2.5 (Ma et al., 2015). Other than NRT/NPF, two homologues of the guard cell S-type anion channel SLAC1, SLAH2 and SLAH3, are also suggested to mediate xylem loading of NO_3_^−^ (Maierhofer et al., 2014a; Maierhofer et al., 2014b). Activation of these two SLAHs is also dependent on CBL1/9-CIPK23, and in addition, several members of the CPK family, such as CPK21 (Maierhofer et al., 2014a; Maierhofer et al., 2014b; Yao et al., 2017). Some members of the NRT/NPF or SLAH family are capable of transporting other ion species as well. NRT1.5/NPF7.3 can mediate K^+^ and NO_3_^−^ loading into the xylem, working synergistically with SKOR to maintain K^+^/NO_3_^−^ balance in root and shoot (Lin et al., 2008; Drechsler et al., 2015; Li et al., 2017b). Another NPF member, NPF2.4, is responsible for Cl^−^ loading into the xylem (Li et al., 2016). SLAH1, a silent channel subunit expressed together with SLAH3 in xylem-pole pericycle cells, mediates root to shoot Cl^−^ translocation by forming a heteromer with SLAH3 (Cubero-Font et al., 2016; Qiu et al., 2016).

Once imported into the xylem, K^+^ travels long-distance from root to shoot to be exported into appropriate aerial tissues. In addition, K^+^ can be transported from green cells into the phloem to be returned back to the roots. Detailed mechanism of this root-shoot translocation of K^+^ still remains ambiguous, although transporters which affect the shoot/root ratio of K^+^ might contribute, such as KUP7 (Han et al., 2016) and OsHAK16 from rice (Feng et al., 2019). Likewise, the identity of the transporters responsible for the retrieval of anions from the xylem remains unclear too, albeit several transporters such as NRT1.8/NPF7.2 (Li et al., 2010; Fan et al., 2017; Zhang et al., 2018) and Cation/Chloride Cotransporters (CCCs) (Li et al., 2017a) have been suggested.

### Regulation of Cell Expansion and Movement

One of the key roles of K^+^, NO_3_^−^, and Cl^−^ in aerial parts of plants is regulation of stomatal aperture. Stomatal movement occurs through change of osmolarity concomitantly with ion flow (mainly K^+^) across the guard cell membrane. A number of guard cell-expressed transporters contribute to this regulation; KAT1, KAT2, AKT1, AKT2, NPF6.3, and H^+^-ATPases such as AHA2 for stomatal opening (Szyroki et al., 2001; Guo et al., 2003; Saito and Uozumi, 2019), and SLAC1, SLAH3, GORK, and ALMT12 for stomatal closing (Hosy et al., 2003; Vahisalu et al., 2008; Meyer et al., 2010; Geiger et al., 2011; Saito and Uozumi, 2019). Stomata, being the site of water loss *via* transpiration and entrance of pathogens, are regulated by specific signal transduction pathways that ensure rapid closure in response to drought or pathogen attack. This signaling is mediated by an increase in guard cell cytosolic Ca^2+^ concentration and the resulting regulation of transporters by activated CPKs or CBL-CIPKs (Pandey et al., 2007b; Munemasa et al., 2015; Saito and Uozumi, 2019). Ca^2+^-activated CPK3, CPK6, CPK21, CPK23 (Geiger et al., 2010; Geiger et al., 2011; Scherzer et al., 2012), and CBL1/9-CIPK23 (Maierhofer et al., 2014a) are capable of eliciting anion efflux through SLAC1 and SLAH3. In addition, CBL5-CIPK11 can also activate SLAC1 (Saito et al., 2018). Following this anion efflux, K^+^ is driven out from guard cells *via* the Shaker K^+^ efflux channel GORK, causing turgor pressure decrease and cell shrinkage, leading to stomatal closure. Moreover, GORK itself, either directly or indirectly, is activated by CPK21 (van Kleeff et al., 2018), CPK33 (Corratgé-Faillie et al., 2017), and CBL1-CIPK5 (Förster et al., 2019). In addition, Ca^2+^ also triggers attenuation of stomatal opening. CIPK11 (although its interacting CBL remains undetermined) has been reported to inhibit AHA2 activity (Fuglsang et al., 2007; Yang et al., 2010), and CPK13 reduces K^+^ influx mediated by the Shaker K^+^ channels KAT1 and KAT2 (Ronzier et al., 2014). Additionally, CBL2/3 and CIPK9/17 were reported to regulate stomatal movement *via* control of vacuolar morphology (Song et al., 2018), possibly achieved by phosphorylation of the vacuolar localized transporters like K^+^/H^+^ antiporter NHX (Barragán et al., 2012; Andres et al., 2014), two pore K^+^ channel TPK1 (Gobert et al., 2007) and V-ATPase (Ratajczak, 2000; Eisenach and De Angeli, 2017). It is noteworthy that *cbl2 cbl3* double mutation in *Arabidopsis* results in reduced activity of V-ATPase (Tang et al., 2012).

Calcium is also well recognized as a predominant regulator of pollen germination and pollen tube elongation in a wide range of plant species (Steinhorst and Kudla, 2013a; Zheng et al., 2019). Control of cell volume through Ca^2+^-dependent regulation of ion channels plays a crucial role in pollen tube growth. So far, CPKs and CBL-CIPKs reported to function in pollen tubes are CPK2/11/17/20/24/34, CBL1/2/3/9, and CIPK12/19 (Myers et al., 2009; Mähs et al., 2013; Zhou et al., 2015). CPK11, together with CPK24, modulates the activity of the pollen-expressed plasma membrane K^+^ influx channel SPIK, which is required for pollen germination (Mouline et al., 2002; Zhao et al., 2013). Pollen tubes also require an anion gradient at the tube tip, which was shown to be maintained by SLAH3 and its activator CPK2 and CPK20 (Gutermuth et al., 2013). CBL2/3-CIPK12 participate in pollen germination and tube growth by controlling vacuole morphology *via* regulation of a yet to be identified tonoplast protein (Steinhorst et al., 2015).

### Sodium and Calcium—Resistance Against Salinity Stress

Sodium (Na^+^) is widely considered the major cause of salt stress damage, osmotic stress, as well as K^+^ deficiency due to their chemical and structural similarity (Benito et al., 2014). Uptake of Na^+^ is likely mediated by non-selective cation channels and considered accidental (Demidchik and Tester, 2002; Keisham et al., 2018). In order to protect leaves and reproductive tissues, plants have developed a sophisticated system for sequestering Na^+^ or sending it back to the soil. The salt overly sensitive (SOS) pathway, a major salt resistance mechanism, is initiated by rapid intracellular Ca^2+^ increase in response to salt treatment (Tester and Davenport, 2003; Köster et al., 2018), suggested to be achieved by opening of Ca^2+^ influx channels by Na^+^-activated GIPC sphingolipids (Jiang et al., 2019). In this pathway, CIPK24 (SOS2), in combination with Ca^2+^-activated CBL4 (SOS3), phosphorylates Na^+^ efflux/H^+^ influx antiporter SOS1 to remove Na^+^ from cells (Qiu et al., 2002; Shi et al., 2002). Alternatively, CBL1-CIPK24 might also mediate this process (Kolukisaoglu, 2004; Manik et al., 2015). Tonoplast localized CBL10 is another CBL required for salt tolerance, presumably by activating a Na^+^/H^+^ antiporter together with CIPK24, allowing compartmentalization of Na^+^ into the vacuole (Kim et al., 2007; Manik et al., 2015). Guard cell K^+^ efflux channel GORK is also expressed in roots. Sudden salt stress induces membrane depolarization and cytosolic Ca^2+^ increase in root cell, which activates GORK *via* a mechanism mentioned earlier in this review. Activated GORK mediates K^+^ efflux from root cells, which alongside with H^+^ efflux by AHA2, repolarizes membrane potential and restores Ca^2+^ homeostasis (van Kleeff et al., 2018).

### Calcium Controlling Ammonium Uptake Level

Plants absorb two kinds of nitrogen species from the soil, nitrate (NO_3_^−^) and ammonium (NH_4_^+^). NO_3_^−^ import and translocation by multiple NRT/NPF and SLAH transporters and regulation of these by Ca^2+^ were described earlier in this review. NH_4_^+^ uptake, on the other hand, is mediated by ammonium transporters (AMT). While NH_4_^+^ is beneficial as an alternative nitrogen source, high levels of NH_4_^+^ can be toxic, and therefore its cellular level must be strictly controlled (Britto and Kronzucker, 2002; Zheng et al., 2015). Two members of the AMT family, AMT1;1 and AMT1;2, were shown to be inhibited by the CBL1-CIPK23 complex, also known as an activator of root-expressed AKT1, HAK5, and SLAH2/3 (Loqué and Von Wirén, 2004; Straub et al., 2017). Thus, it is likely that CBL1-CIPK23 plays a key role in maintaining ion homeostasis in root cells and in preventing the toxic effects of NH_4_^+^ (Britto and Kronzucker, 2002; Zheng et al., 2015). Another element that controls the NO_3_^−^/NH_4_^+^ balance is the transcription factor NLP7, which was recently shown to induce up-regulation of NPF6.3 transcripts in the presence of NH_4_^+^ (Zhao et al., 2018b). NLP7 is phosphoregulated by CPK10/30/32, which are activated by NO_3_^−^-dependent elevation of intracellular Ca^2+^ (Liu et al., 2017).

### Possible Role of Calcium in Balancing of Energy Source

Essential nutrients translocated through vascular tissues are not only limited to ions but also include organic compounds such as amino acids and sugars (Fischer et al., 1998; Liu et al., 2012a). Sugars are transported through phloem in the form of sucrose and distributed throughout the plant (Liu et al., 2012a). Loading of sucrose from the phloem to the apoplast requires activity of the shaker K^+^ channel AKT2 (Shabala, 2003; Dreyer et al., 2017; Ragel et al., 2019). AKT2, usually weakly-rectified, can be converted into a non-rectifying K^+^ channel *via* phosphorylation, thereby enabling K^+^ efflux and phloem membrane repolarization and the consequent retrieval of sucrose (Deeken et al., 2002; Michard et al., 2005a; Michard et al., 2005b; Gajdanowicz et al., 2011; Sandmann et al., 2011; Saito et al., 2017). Though the kinase responsible for this phosphorylation remains to be identified, it must be noted that AKT2 activity can be enhanced by the CBL4-CIPK6 complex in a Ca^2+^-dependent but phosphorylation-independent manner (Held et al., 2011).

The efficiency of cellular energy use is optimized by carbon/nitrogen (C/N) balance, and therefore its maintenance is of great significance for growth and development of plants (Coruzz and Bush, 2001; Zheng, 2009; Maekawa et al., 2014). In a recent study, three members of CIPK, CIPK7/12/14, were identified as key regulators of the C/N-nutrient response, achieved through their phosphorylation of ubiquitin ligase ATL31 (Yasuda et al., 2014; Yasuda et al., 2017).

Additionally, although most of the carbon compounds are derived from photosynthesis, plants respond to externally supplied sugars as well. These exogenous sugars, in addition to their use as energy source, show hormone-like behavior, working in parallel with some of the ABA-responsive genes (Rolland and Sheen, 2005; Yamada et al., 2011; Singh et al., 2014; Williams et al., 2014; Yuan et al., 2014). Among the calcium-regulated phosphorylation modules, CBL1 (Li et al., 2013) and CIPK14 (Yan et al., 2014) were found to positively regulate the response to glucose by an yet unidentified mechanism.

### Uptake Regulation of Metal Ions and Toxins

Metal ions such as magnesium (Mg^2+^), iron (Fe), zinc (Zn^2+^), and manganese (Mn^2+^) work as cofactors of numerous enzymes and are therefore indispensable for plant growth. Several Ca^2+^-regulated phosphorylation components also participate in maintaining homeostasis of these ions. Iron deficiency was reported to elicit an increase of Ca^2+^ in *Arabidopsis* roots. This induces CBL1/9-CIPK23 to enhance Ferric chelate reductase (FRO) activity, which is required for converting Fe^3+^ in the soil into the transported form, Fe^2+^, thereby substantially regulating iron acquisition (Tian et al., 2016). CIPK23 alongside with CBL2/3 and CIPK3/9, is also required for modulation of plant growth under high Mg^2+^ condition, likely mediated by Mg^2+^ compartmentalization to the vacuole (Mogami et al., 2015; Tang et al., 2015). Additionally, Zn^2+^ and Mn^2+^ levels were found to be reduced in *cipk23* mutant plants, suggesting some unidentified regulatory system of metal acquisition involving CIPK23 (Tian et al., 2016). On the other hand, some CPKs and CBL-CIPKs are involved in uptake of toxic ions. For instance, CPK31 was reported to regulate uptake of non-essential and toxic arsenite (As^3+^) (Ji et al., 2017), and in rice, several members of the CPK family exhibited increased phosphorylation in response to cadmium (Cd^2+^) application (Zhong et al., 2017).

## Conclusions

**Figure 1:**
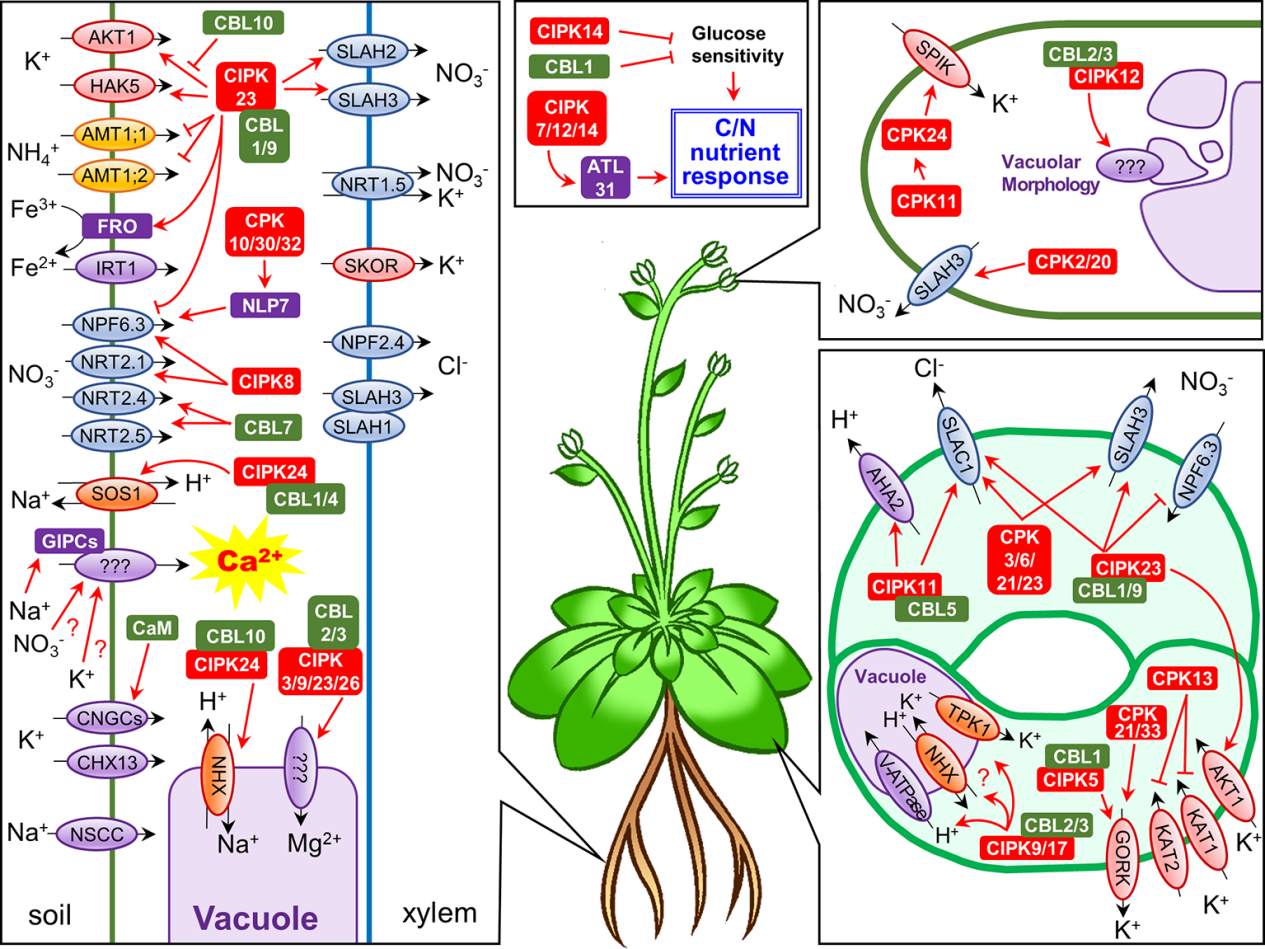
Schematic representation of Ca^2+^-regulated nutrient uptake and translocation in *Arabidopsis thaliana*. Each panel shows ion channel/transporter regulation by Ca^2+^-dependent phosphorylation systems in roots, guard cells, and pollen tubes, or the roles of CBLs/CIPKs in C/N nutrient response, respectively. Abbreviations: NSCC, non-selective cation channel; IRT1, iron transporter 1; CaM, calmodulin.

In this minireview we have summarized the Ca^2+^-regulated uptake, storage, and translocation of nutrient ions, and possible role of Ca^2+^ in energy source balancing (**Figure 1**, **Table 1**). Most of these regulatory mechanisms are initiated by a rise of the cytosolic Ca^2+^ level in response to stress or nutrient depletion, and ultimately lead to resistance against unfavorable conditions. Thus, full understanding of the Ca^2+^-dependent phosphorylation machinery would be a vital step for optimizing plant growth and reproduction.

**Table 1:** Summary of ion channels/transporters and other Ca^2+^-regulated proteins reviewed in this article.

Name	Activator	Deactivator	Type of transport	Expressed in	Role
AKT1	CBL1/9-CIPK23	CBL10	K^+^ influx	Root, guard cell	K^+^ uptake, stomatal opening
					
HAK5	CBL1/9-CIPK23		K^+^ influx	Root	K^+^ uptake
					
Unknown	CBL2/3-CIPK9				K^+^ homeostasis
CNGC3	Calmodulin?		K^+^ influx	Root	K^+^ uptake
CNGC10	Calmodulin?		K^+^ influx	Root	K^+^ uptake
CNGC13	Calmodulin?		K^+^ influx	Root	K^+^ uptake
CHX13			K^+^ influx	Root	K^+^ uptake
					
SKOR			K^+^ efflux	Root xylem pericycle	Xylem loading of K^+^
					
NRT2.1	CIPK8		NO_3_^−^ influx	Root	NO_3_^−^ uptake
NRT2.2			NO_3_^−^ influx	Root	NO_3_^−^ uptake
NRT2.4	CBL7		NO_3_^−^ influx	Root	NO_3_^−^ uptake
NRT2.5	CBL7		NO_3_^−^ influx	Root	NO_3_^−^ uptake
NPF2.3			NO_3_^−^ influx	Root	NO_3_^−^ uptake
NPF4.6			NO_3_^−^ influx	Root	NO_3_^−^ uptake
					
NPF6.3	CIPK8, CPK10/30/32 (via NLP7 phosphorylation)	CBL1/9-CIPK23(via conversion of NO_3_^−^ affinity mode)	NO_3_^−^ influx	Root, guard cell	NO_3_^−^ uptake, stomatal opening
					
NRT1.5			K^+^/H^+^ antiport, NO_3_^−^ efflux	Root xylem pericycle	Xylem loading of K^+^ and NO_3_^−^
					
NPF2.4			Cl^−^ efflux	Root xylem pericycle	Xylem loading of Cl^−^
					
SLAH2	CBL1/9-CIPK23		NO_3_^−^ efflux	Root stele	Xylem loading of NO_3_^−^
					
SLAH3	CBL1/9-CIPK23, CPK3/6/21/23, CPK2/20		NO_3_^−^ efflux, Cl^−^ efflux (when forming heteromer with SLAH1)	Root xylem pericycle, guard cell, pollen tube	Xylem loading of NO_3_^−^ and Cl^−^, stomatal closure, pollen tube elongation
					
AKT2	CBL4-CIPK6		Weak/non-rectified K^+^ transport (switched by phosphorylation)	Phloem, guard cell	Phloem membrane repolarization
					
KAT1		CPK13	K^+^ influx	Guard cell	Stomatal opening
					
KAT2		CPK13	K^+^ influx	Guard cell	Stomatal opening
					
AHA2		CIPK11	H^+^ efflux	Guard cell	Stomatal opening
					
GORK	CPK21/33, CBL1-CIPK5 (via inhibition of ABI2)		K^+^ efflux	Root, guard cell	Restoring root Ca^2+^ homeostasis, stomatal closure
					
SLAC1	CBL1/9-CIPK23, CPK3/6/21/23, CBL5-CIPK11		Cl^−^ efflux	Guard cell	Stomatal closure
					
ALMT12			Malate efflux	Guard cell	Stomatal closure
					
Unknown	CBL2/3-CIPK9/17			Guard cell tonoplast	Control of guard cell vacuolar morphology?
					
V-ATPase	CBL2/3-unidentified CIPK		H^+^ influx	Guard cell tonoplast	Vacuolar pH homeostasis
					
SPIK	CPK11 and CPK24 together		K^+^ influx	Pollen tube	Pollen tube growth
					
SOS1	CBL1/4-CIPK24		Na^+^/H^+^ antiport	Root	Removal of Na^+^ from root cell
					
Unknown	CBL10-CIPK24		Na^+^/H^+^ antiport	Root vacuole	Na^+^ compartment into vacuole
					
Unknown	Na^+^ bound GIPC		Ca^2+^ influx	Root	Initiation of cytosolic Ca^2+^ increase
					
AMT1;1		CBL1/9-CIPK23	NH_4_^+^ influx	Root	NH_4_^+^ uptake
					
AMT1;2		CBL1/9-CIPK23	NH_4_^+^ influx	Root	NH_4_^+^ uptake
					
Unknown	CBL1, CIPK14		Glucose?		Glucose response
					
ATL31 (ubiquitin ligase)	CIPK7/12/14	–	–	Ubiquitous	Regulation of C/N-nutrient response
					
FROs (ferric chelate reductase)	CBL1/9-CIPK23	–	–	Varies	Iron acquisition
					
Unknown	CBL2/3-CIPK3/9/23			Tonoplast	Mg^2+^ storage
					
NIP1;1	CPK31		As^3+^ influx	Root	As^3+^ uptake

## Author Contributions

Conceptualization, original draft preparation, review, and editing were done by SS, and NU contributed to the article by funding acquisition.

## Funding 

This work was supported by JSPS KAKENHI Grant Number (16H06558, 18H03762, 19H02880, 19K21140, and 19K22264).

## Conflict of Interest

The authors declare that the research was conducted in the absence of any commercial or financial relationships that could be construed as a potential conflict of interest.
